# Thiophene Disubstituted Benzothiadiazole Derivatives: An Effective Planarization Strategy Toward Deep-Red to Near-Infrared (NIR) Organic Light-Emitting Diodes

**DOI:** 10.3389/fchem.2019.00276

**Published:** 2019-04-18

**Authors:** Wentao Xie, Binbin Li, Xinyi Cai, Mengke Li, Zhenyang Qiao, Xiaohui Tang, Kunkun Liu, Cheng Gu, Yuguang Ma, Shi-Jian Su

**Affiliations:** State Key Laboratory of Luminescent Materials and Devices, Institute of Polymer Optoelectronic Materials and Devices, South China University of Technology, Guangzhou, China

**Keywords:** organic light-emitting diodes, donor-acceptor chromophores, deep-red to near-infrared (NIR) emission, hybridized local and charge-transfer state (HLCT), hot-exciton

## Abstract

As one of the three primary colors that are indispensable in full-color displays, the development of red emitters is far behind the blue and green ones. Here, three novel orange-yellow to near-infrared (NIR) emitters based on 5,6-difluorobenzo[c][1,2,5]thiadiazole (BTDF) namely BTDF-TPA, BTDF-TTPA, and BTDF-TtTPA were designed and synthesized. Density functional theory analysis and photophysical characterization reveal that these three materials possess hybridized local and charge-transfer (HLCT) state feature and a feasible reverse intersystem crossing (RISC) from the high-lying triplet state to the singlet state may conduce to an exciton utilization exceeding the limit of 25% of traditional fluorescence materials under electrical excitation. The insertion of thiophene with small steric hindrance as π-bridge between the electron-donating (D) moiety triphenylamine (TPA) and the electron-accepting (A) moiety BTDF not only results in a remarkable 67 nm red-shift of the emission peak but also brings about a large overlap of frontier molecular orbitals to guarantee high radiative transition rate that is of great significance to obtain high photoluminescence quantum yield (PLQY) in the “energy-gap law” dominated long-wavelength emission region. Consequently, an attractive high maximum external quantum efficiency (EQE) of 5.75% was achieved for the doped devices based on these thiophene π-bridged emitters, giving a deep-red emission with small efficiency roll-off. Remarkably, NIR emission could be obtained for the non-doped devices, achieving an excellent maximum EQE of 1.44% and Commission Internationale de l'Éclairage (CIE) coordinates of (0.71, 0.29). These results are among the highest efficiencies in the reported deep-red to NIR fluorescent OLEDs and offer a new π-bridge design strategy in D-π-A and D-π-A**-**π-D red emitter design.

## Introduction

Since the creative invention of organic light-emitting diodes (OLEDs) by Tang and VanSlyke (1987), OLEDs have been receiving intense research for more than 30 years for the potential applications in flat panel display (Pfeiffer et al., 2002), solid state lighting (Kido et al., 1995) and other applications outside the visible range (Tessler et al., 2002). According to spin statistics rule, the branching ratio of singlet and triplet excitons is 1:3, meaning one singlet exciton is generated for every three triplet excitons under electrical excitation (Baldo et al., 1999). Therefore, since radiative transition of triplets is spin-forbidden, internal quantum efficiency (η_int_) is limited to 25% for traditional fluorescence. Transition metals such as iridium and platinum were introduced into organic aromatic frameworks by Baldo et al. (1998), to harvest triplets by increasing spin-orbit coulping (SOC) between the first singlet excited state (S_1_) and the first triplet excited state (T_1_), and nearly 100% internal quantum efficiency can be theoretically obtained. However, the usage of noble metals like Ir and Pt are expensive and non-renewable, which are fatal to large area, low cost production in future. Hence, searching for high efficiency and noble metal-free purely organic emitting materials is imperative (Chen et al., 2016).

Recently, many purely organic luminescence mechanisms with internal quantum efficiency over 25% were proposed by different research groups around the world, such as thermally activated delayed fluorescence (TADF) (Uoyama et al., 2012), triplet–triplet annihilation (TTA) (Kondakov et al., 2009), hybridized local and charge-transfer state (HLCT) (Li et al., 2012) and neutral π radical doublet emission (Peng et al., 2015). Among these luminescence mechanisms, TADF materials were regarded as the most promising next generation luminescent materials for their fascinating advantages: (1) concise design concept (Cai et al., 2016b); (2) tunable full spectrum emission (Park et al., 2016); and (3) high efficiency that can rival those of phosphorescent OLEDs (Liu M. et al., 2017). Unfortunately, TADF-OLEDs are often sufferred from severe efficiency roll-off at high current density due to triplet–triplet annihilation (TTA) and triplet–polaron annihilation (TPA) induced by their long triplet exciton lifetimes, regardless of high EQEs at low current density (Zhang et al., 2012). Yet, the most essential reason is the small radiative transition rate (*k*_*f*_) of these TADF materials (Chen et al., 2019). As one of the three primary colors that are indispensable in full-color display, the research progress in red OLEDs still lags behind the development of highly efficient blue and green OLEDs (Lin et al., 2016; Wu et al., 2018). The reason for the slow development of red luminescent materials can be attributed to the “energy-gap law,” i.e., when the energy gap decreased, the coupling (or vibrational overlap) between the zero-vibrational level of S_1_ and the higher levels of S_0_ state is enhanced (Furue et al., 2018). As a result, the red luminescent materials often sufferred from the accelerated energy loss caused by the enhanced non-radiative internal conversion (IC) (Chen et al., 2018). Therefore, the common large twist donor-acceptor structure in blue and green TADF-OLEDs is not suitable for the design of highly efficient red OLEDs with small efficiency roll-off due to the small *k*_*f*_.

**Scheme 1:**
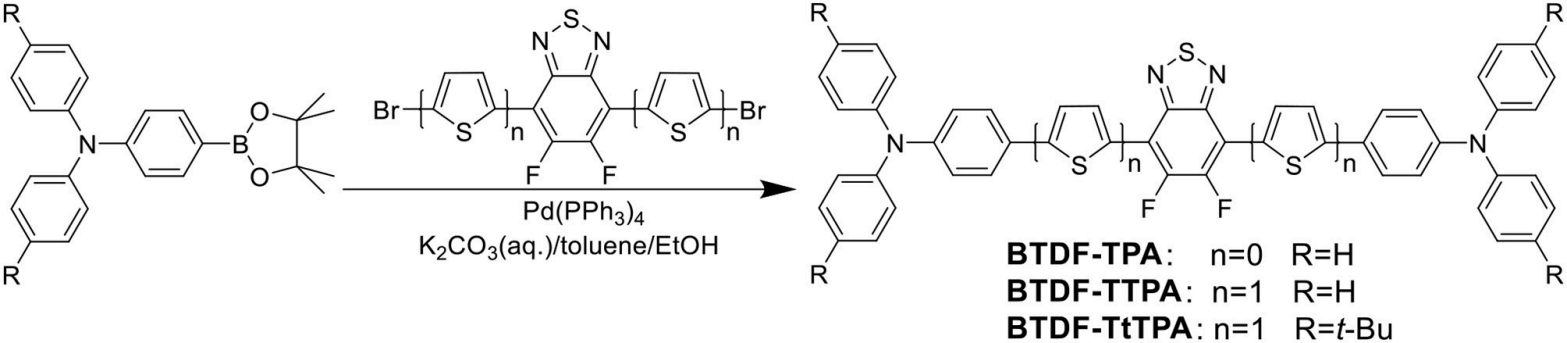
**Synthetic route of the investigated molecules**.

Fortunately, the HLCT state is likely suitable for the design of red emission materials, where the locally excited (LE) state contributes to a high photoluminescence (PL) efficiency and the charge-transfer (CT) state contributes to a large fraction of triplet exciton utilization in electroluminescence (EL) (Li et al., 2014b). Therefore, to achieve a highly efficient and small efficiency roll-off deep-red to NIR purely organic luminescent materials, three BTDF-based compounds were designed and synthesized (Scheme 1 and Figure S1). By incorporating a thiophene unit with small steric hindrance as π-bridge in the common D-A-D skeleton, a remarkable 67 nm red-shift of the emission peak was achieved with the maintenance of high radiative transition rate that is of great significance to obtain high PLQY in “energy-gap law” dominated long-wavelength emission region (Zhang et al., 2014).

## Results and Discussion

### Theoretical Calculation

**Figure 1:**
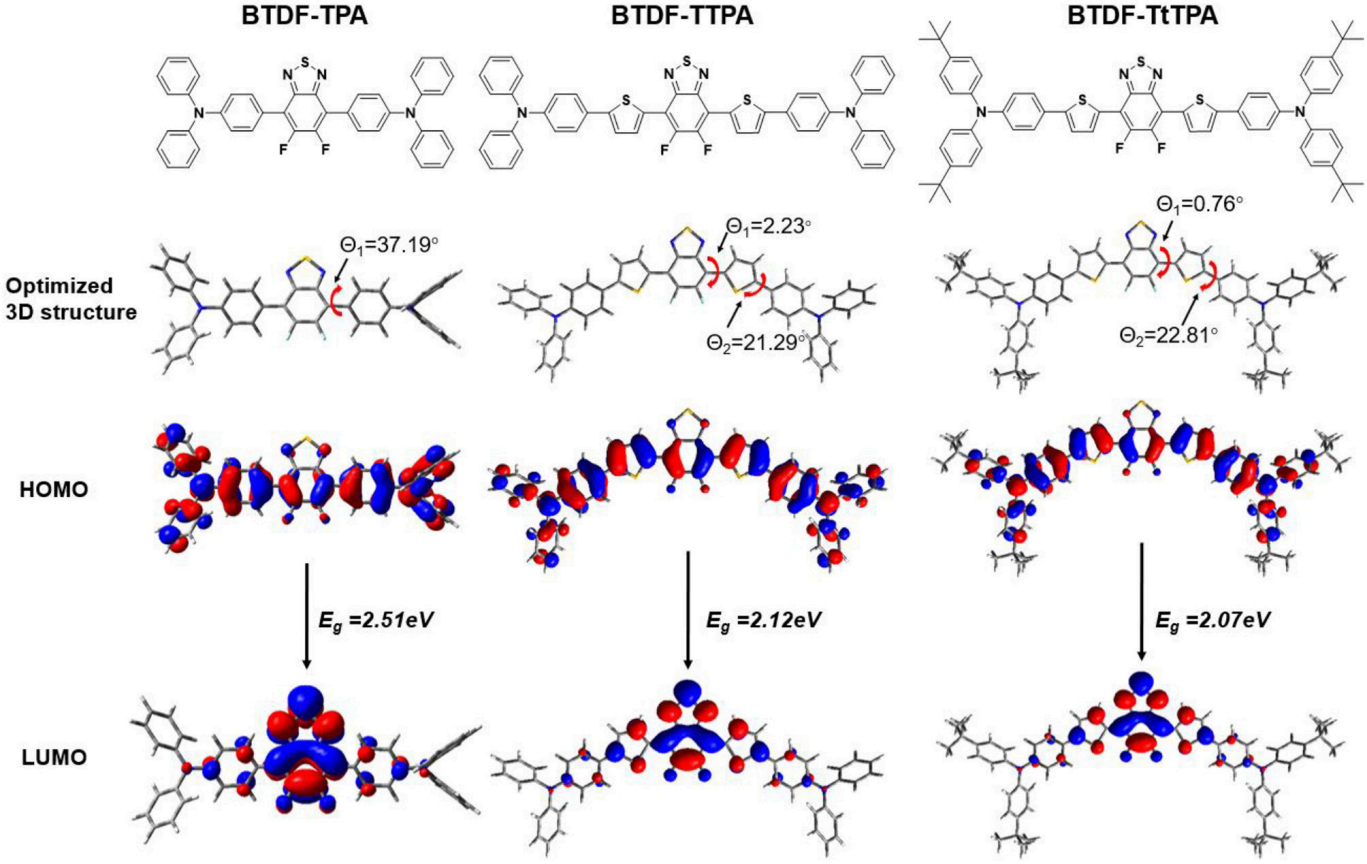
Optimized 3D structure and frontier molecular orbital distributions of BTDF-TPA, BTDF-TTPA, and BTDF-TtTPA.

**Figure 2:**
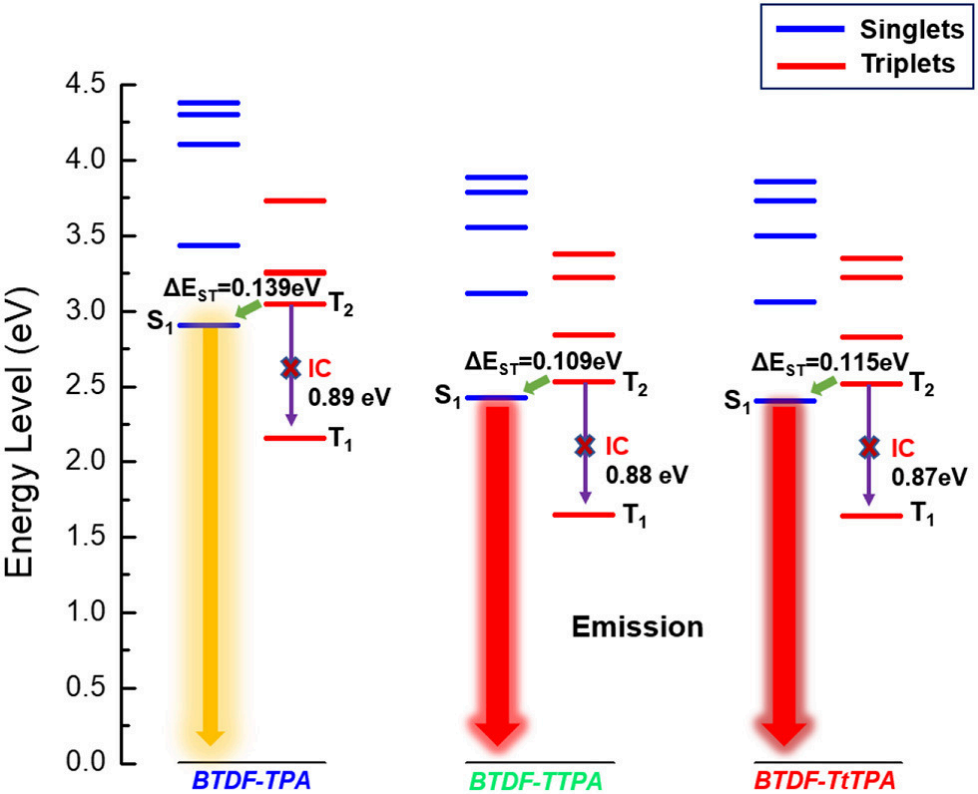
The energy landscape of singlet and triplet excited states for the BTDF-based compounds.

In order to explore the relationship between the material property and molecular structure of these compounds, density functional theory (DFT) simulation was performed firstly using the Gaussian suite of programs (Gaussian 09-B01 package). The ground-state geometries of the investigated compounds were optimized at the B3LYP/6-31G (d, p) level, and geometries and frontier molecular orbital (FMO) distributions were depicted in Figure 1. As shown, the highest occupied molecular orbitals (HOMOs) of these compounds are distributed throughout the whole molecular skeleton, but the lowest unoccupied molecular orbitals (LUMOs) are mainly located on the BTDF acceptor and slightly extended to the benzene or thiophene π-bridges. The large overlaps of HOMOs and LUMOs imply a decent radiative transition rate for these materials to achieve high PLQY (Gan et al., 2018). Comparing BTDF-TTPA (or BTDF-TtTPA) with BTDF-TPA, the introduction of the five-membered ring thiophene bridge between the electron donor (D) and the electron acceptor (A) not only extends the conjugation length of the whole molecule to ensure a red-shift of the emission wavelength, but also reduces the dihedral angle between D and A by a wide margin to obtain a large oscillator strength for the sake of moderate PLQY (Cai et al., 2016a). Futhermore, to deeply describe the excited state properties of these investigated materials, natural transition orbitals (NTOs) and the energy levels of singlets (S_1_ to S_5_) and triplets (T_1_ to T_5_) were calculated at the level of TD-M062X/6-31G (d, p) on the basis of the optimized S_0_ state configuration (Figure 2 and Figure S2) (Han et al., 2015). For the lowest singlet excited states (S_1_), the particles are obviously localized on the BTDF acceptor component, but the holes are dispersed on the whole molecules. Therefore, a majority of LE transition of BTDF and a minority of CT transition from TPA to BTDF can be predicted. The overlap of holes and particles demonstrated the coexistence of LE and CT components, implying the existence of the HLCT state, which was proposed by Li et al. (2014a) in recent years. However, the lowest triplet excited states (T_1_) were a LE state, and its holes and particles were localized on the BTDF acceptor moiety and almost completely overlapped. The configuration of the high-lying triplet excited states T_2_ is quite similar to that of the S_1_ states, indicating the T_2_ states also possess the HLCT feature. The coexistence of the LE and CT states in the S_1_ and T_2_ states are in favor of the reverse intersystem crossing (RISC) according to the permissable SOC betwen the singlet state and the triplet state, and the RISC rate can be greatly enhanced since the sulfur atoms in the thiophene and BTDF heterocyclics are able to improve SOC (Yao et al., 2014). As depicted in Figure 2, a significantly large energy gaps (0.87–0.89 eV) between T_2_ and T_1_ may inhibit the IC from T_2_ to T_1_ according to the energy-gap law, and the small energy split (0.109–0.139 eV) between T_2_ and S_1_ may facilitate the RISC from T_2_ to S_1_ (Liu T. et al., 2017). Such energy level landscape meets the requirement of “hot exciton” mechanism very well, leading to a high-lying RISC process from T_2_ to S_1_, and an exciton utilization exceeding 25% under electrical excitation can be expected (Tang et al., 2018).

### Photophysical Properties

**Figure 3:**
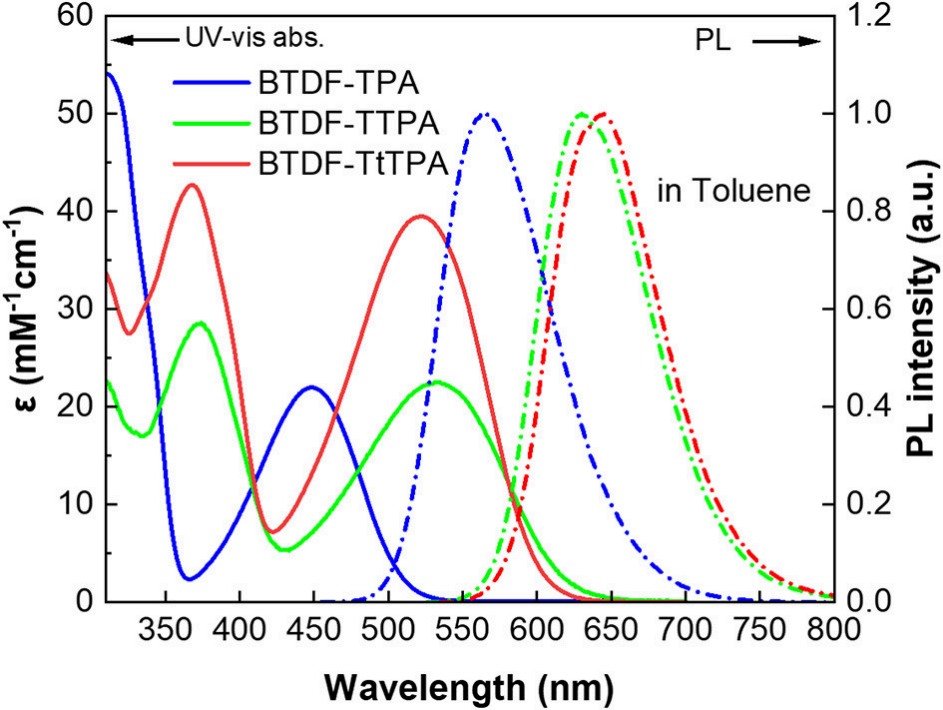
UV-vis absorption (solid lines) and photoluminescence (PL) (dashed lines) spectra of the investigated molecules in toluene solution (10^−5^ M) at room temperature.

To clarify the photophysical properties of the BTDF-based compounds, ultra-violet and visible (UV-vis) absorption and PL spectra were firstly measured in different polar solvents (Figure 3, Figure S6 and Table S2). As depicted in Figure 3, the short-wavelength absorption bands at around 325 and 375 nm could be associated with π-π^*^ transition, while the weak absorption bands at around 375–525 and 425–625 nm could be attributed to the intramolecular charge-transfer (ICT) transition from the TPA moiety to the BTDF moiety (Tsai et al., 2015). As the polarity of the solvent increases (Figure S6), the absorption spectra change slightly in shape, which means that the dipoles change in the ground state is quite small in different polar solvents (Yao et al., 2014). Furthermore, despite the introduction of the thiophene bridge results in about 67 nm red shift of the emission spectra, the molar extinction coefficients (ε) of the ICT transitions in toluene solution still remain a relatively high level (about 2–4 × 10^4^ L mol^−1^ cm^−1^), which should most likely be ascribed to the effective planarization molecular design strategy (Jiang et al., 2017). The emission colors of these materials in dilute toluene solution (10^−5^ M) are in a range from orange-yellow (BTDF-TPA: λ_em_ = 563 nm) to deep-red (BTDF-TTPA: λ_em_ = 630 nm; BTDF-TtTPA: λ_em_ = 645 nm). Besides, the PL spectra of these BTDF-based compounds in different polar solvents show a significant red-shift phenomenon as the solvent polarity increases: the emission peak wavelength moves from 605 nm in low polar solvent n-hexane to 689 nm in high polar solvent acetone for BTDF-TTPA. These remarkable solvatochromic phenomena in solution indicate that the excited states of these investigated compounds have a strong CT characteristic and the dipoles change a lot (Zhao et al., 2018). Considering the emissions of BTDF-TTPA and BTDF-TtTPA in toluene solution are located in the deep-red region, UV-vis absorption and PL spectra of these two materials in neat thin film have also been characterized (Figure S3). They exhibit an emission peak of 663 nm, indicating the possibility to fabricate a non-doped NIR device.

**Figure 4:**
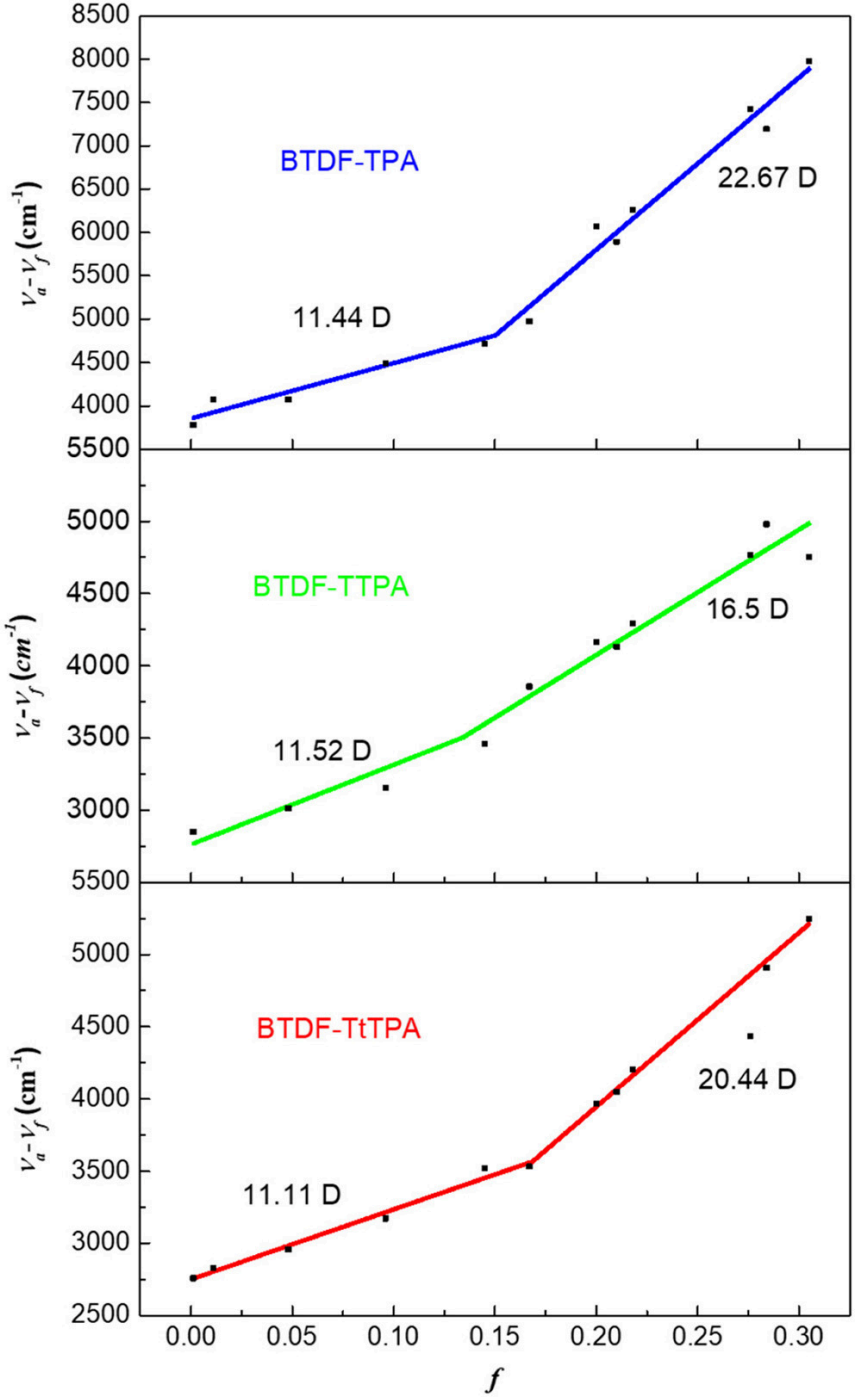
Linear fitting of the Lippert–Mataga model for BTDF-TPA, BTDF-TPA, and BTDF-TtTPA (*f*, orientation polarization of solvent media; *v*_*a*_ − *v*_*f*_, Stokes shift).

Moreover, in order to deeply understand the relationship between the excited state properties and the solvent polarity, the dipole moment of the excited state (μ_e_) on the basis of the Lippert–Mataga relation was measured for these three compounds. As shown in Figure 4, the linear relation of the Stokes shift (ν_a_-ν_f_) vs. the orientation polarizability *f*(ε, *n*) was fitted for BTDF-TPA, BTDF-TTPA, and BTDF-TtTPA. They all displayed two different linear relations in low-polarity and high-polarity region, respectively. Taken BTDF-TPA as an example, the dipole moment μ_e_ of 11.44 Debye in low polar solvents indicate a LE-state dominated character, while the μ_e_ of 22.67 Debye in high polar solvents can be attributed to CT-state. Based on the above analysis, the inter-crossing and the coexistence of LE and CT components can facilitate the HLCT state in medium polarity solvents for the BTDF-based compounds (Li et al., 2014a).

Transient PL decay characteristics of these compounds were also investigated in toluene solution, doped film and neat film (Figure S5 and Table S1). The transient PL decay curves of these materials in toluene solution exhibit a single-exponential fluorescence decay process with a lifetime of 1.28, 0.96 and 1.10 ns for BTDF-TPA, BTDF-TTPA, and BTDF-TtTPA, respectively. No delayed fluorescence was observed from the transient PL decay curves, suggesting these compounds are not TADF emitters. With the combination of the PLQY in toluene solution, radiative rate constants could be calculated as 7.4 × 10^8^, 8.9 × 10^8^, and 7.5 × 10^8^ s^−1^ for BTDF-TPA, BTDF-TTPA, and BTDF-TtTPA, respectively, by the equation $k_{f}=\frac{1}{\tau_{f}}$. Obviously, throughout the above analysis one can know that the effective thiophene-bridge planarization strategy not only results in a remarkable red-shift of the emission peak but also brings about a large overlap of frontier molecular orbitals to guarantee high radiative transition rate that is of great significance to obtain high PLQY in the “energy-gap law” dominated long-wavelength emission region.

### Thermal Properties and Electrochemical Characterization

As a good thermal stability helps to form an uniform evaporated amorphous film during the fabrication of OLED devices, these compounds were also subjected to thermal analysis by differential scanning calorimetry (DSC) and thermogravimetry (TG) (Zhang et al., 2017). Figure S7 shows the TG and DSC curves of these compounds. High decomposition temperatures (T_d_) from 453 to 493°C were found for the materials corresponding to 5% mass loss. Different from BTDF-TPA and BTDF-TtTPA that show no glass transition temperature (T_g_) and crystallization temperature (T_c_) in the range of 50–200°C, BTDF-TTPA exhibits a T_g_ at 117°C and a clear T_c_ at 161°C. The observed T_g_ and T_c_ for BTDF-TTPA can be attributed to the more planar molecular structure and closer molecular packing due to the introduction of the small steric thiophene as the bridge and the absence of *tert*-butyl substituent group (Qian et al., 2009).

Electrochemical properties of these materials were obtained by cyclic voltammetry (CV) measurement. The HOMO energy levels of BTDF-TPA, BTDF-TTPA, and BTDF-TtTPA were estimated as −5.32, −5.03, and −5.06 eV, respectively. The oxidation potentials were obviously lowered by the introduced electron-rich thiophene bridge, as clearly presented in Figure S8. With the combination of the band-gap obtained from the absorption edge in toluene solution, their LUMO energy levels were estimated to be −2.89, −2.97, and −3.05 eV, respectively. Considering the oxidation potential was measured in a solution, which may be somewhat different from the film state in devices, ionization potentials of BTDF-TTPA (5.30 eV) and BTDF-TtTPA (5.12 eV) in a neat film were also measured by photoelectron yield spectroscopy (Figure S9). Their electron affinities were estimated by adding the corresponding optical energy gaps (E_g_), which were determined from the onset of the neat film absorption spectra (Figure S3). The key parameters of the BTDF-based compounds are summarized in Table 1.

**Table 1:** Photophysical, thermal, and electrochemical properties of the investigated compounds.

**Compounds**	****λ_abs_** [nm]^**a**^**	****λ_em_** [nm]^**a**^**	**T_**d**_/T_**g**_ [^**°**^C]^***b***^**	**HOMO/LUMO/E_**g**_ [eV]^**c**^**	****ϕ_PL_** [%]^**d**^**
BTDF-TPA	310, 451	563	453/N.A.	5.32/2.89/2.43	95.2
BTDF-TTPA	368, 524	630	490/124	5.03/2.97/2.06	85.8
BTDF-TtTPA	371, 531	645	493/N.A.	5.06/3.05/2.01	82.9

a *UV–vis absorption and PL spectra measured in toluene solution (10^−5^ M) at room temperature*.

b *Decomposition (T_d_) (at 5% weight loss) and glass transition temperatures (T_g_)*.

c *HOMO energy levels calculated from the empirical formula: E_HOMO_ = –(E_ox_+4.4 eV), LUMO energy levels estimated from the absorption edge in toluene solution (λ_onset_) and E_HOMO_, using empirical formula: E_LUMO_ = E_HOMO_+ 1,240/λ_onset_*.

d *PL quantum yields were measured in toluene solution (10^−5^ M) at room temperature using an integrating sphere*.

### OLED Characterization

**Figure 5:**
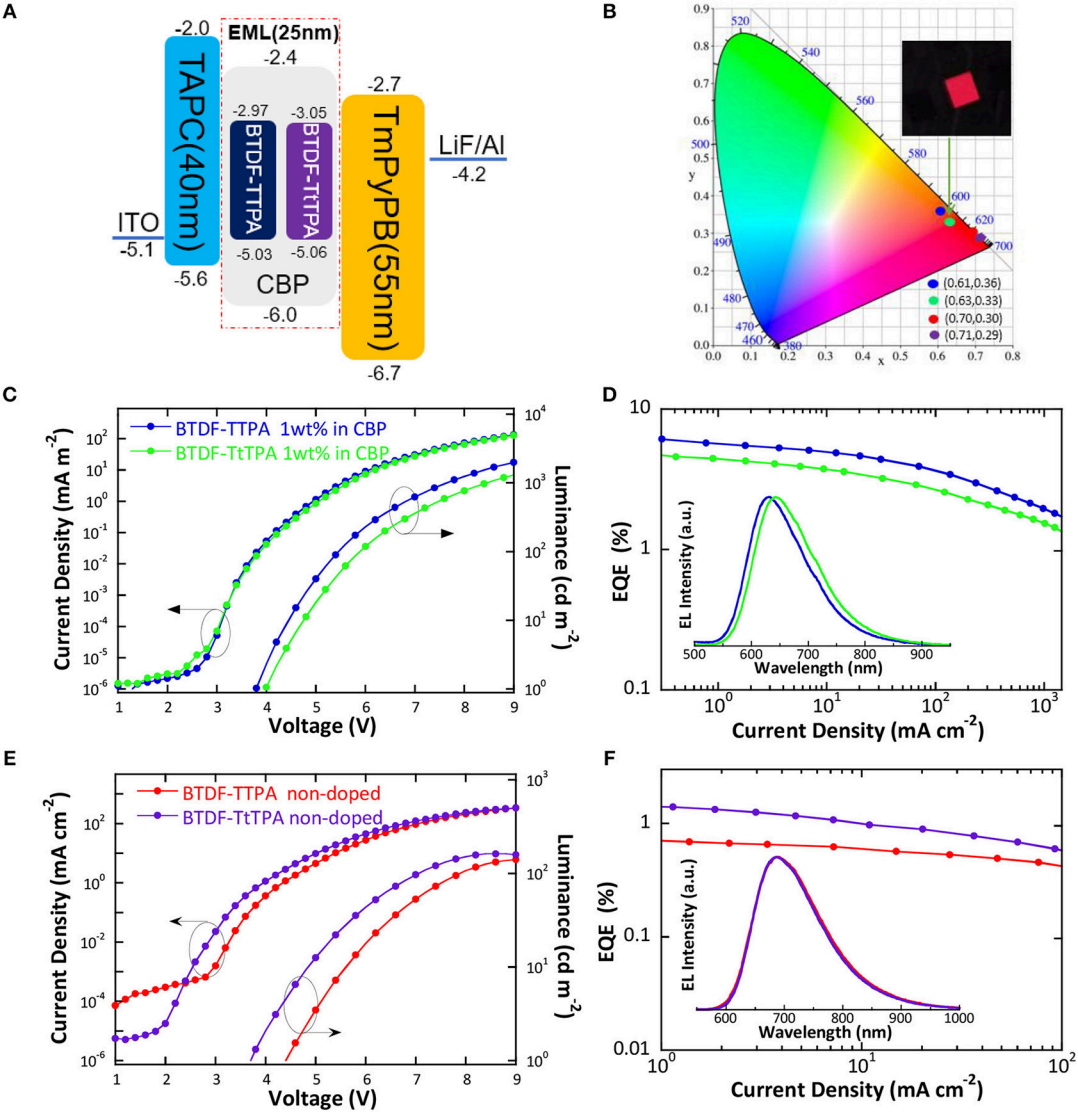
**(A)** Schematic energy level diagram of the doped and non-doped devices based on BTDF-TTPA and BTDF-TtTPA; **(B)** CIE coordinates of the doped and non-doped devices using BTDF-TTPA and BTDF-TtTPA as the emitter at the current density of 1 mA cm^−2^ (the inset photograph is the device of BTDF-TtTPA 1 wt.% in CBP); **(C)** Current density-voltage-luminance and **(D)** external quantum efficiency-current density characteristics of the doped devices (Inset: EL spectra of the doped devices at the current density of 1 mA cm^−2^); **(E)** Current density-voltage-luminance and **(F)** external quantum efficiency-current density characteristics of the non-doped devices (Inset: EL spectra of the non-doped devices at the current density of 1 mA cm^−2^).

According to the PL spectra of these materials in toluene solution, BTDF-TTPA and BTDF-TtTPA are promising deep-red and NIR emitters for OLED applications. Firstly, in order to avoid aggregation caused quenching (ACQ) which may affect device performance, BTDF-TTPA and BTDF-TtTPA were used as dopants dispersed in a common host CBP (4,4′-bis(carbazol-9-yl) biphenyl) in a concentration of 1 wt.% (Xie et al., 2016). As depicted in Figure 5A, a simple device architecture of indium tin oxide (ITO)/TAPC (40 nm)/EML (25 nm)/TmPyPB (55 nm)/LiF (1 nm)/Al (100 nm) was fabricated, in which 1,1-bis(4-(*N*,*N*-di(*p*-tolyl)-amino)-phenyl)cyclohexane) (TAPC), 1,3,5-tri(*m*-pyrid-3-ylphenyl)benzene (TmPyPB) and LiF play the roles of hole-transport and electron-blocking, electron-transport and hole-blocking and electron-injection layers, respectively (Figure S10). Meanwhile, a non-doped emission layer (EML) was also adopted in the same device structure to obtain NIR emissions. As the HOMO and LUMO energy levels of all guest molecules are shallower and deeper than that of the host (CBP), relatively equilibrium hole and electron capture abilities can be anticipated.

The results of the EL performance are recorded in Figures 5C–F, including current density-voltage-luminance (J-V-L), external quantum efficiency (EQE) vs. current density curves for the devices, and the key device parameters are summarized in Table 2. All the devices displayed EL spectra similar to the corresponding PL spectra in doped or neat films, confirming the EL emission was generated solely from the developed emitters (Figures S3, S4). The doped devices exhibit excellent deep-red emission with the emission peaks of 630 and 642 nm, and the device based on 1 wt.% BTDF-TtTPA in CBP shows a Commission Internationale de l'Éclairage (CIE) coordinates of (0.63, 0.33) (Figure 5B), which is quite close to the standard red of (0.67, 0.33) defined by the National Television System Committee (NTSC). A maximum EQE of 5.75% was achieved with small efficiency roll-off, which is among the highest device performance in the reported red fluorescent OLEDs. Besides, the non-doped devices of these two materials appear the same NIR EL emission with a maximum wavelength (λ_el_) of 690 nm and CIE coordinates of (0.70, 0.30) and (0.71, 0.29) (Figure 5B). Although the emission of the non-doped devices is in the NIR range, the highest EQE value of 1.44% obtained by BTDF-TtTPA is also one of the best device performances at the same emission wavelength.

**Table 2:** EL performance of the doped and non-doped devices using BTDF-TTPA and BTDF-TtTPA as emitters.

**EMLs**	**V${\text{V}}_{\text{on}}^{\text{a}}$**	**EQE (%) / CE (cd A**^****−1****^**)**/**PE (lm W**^****−1****^**)**			**Luminance**	**CIE^**b**^**
		**Max**	**@100 mA cm^**−2**^**	**@200 mA cm^**−2**^**	**(cd m^**−2**^)**	
BTDF-TTPA 1 wt.% in CBP	3.8	5.75/5.15/4.27	1.54/1.67/0.58	1.00/1.10/0.33	2,644	(0.61, 0.36)
BTDF-TtTPA 1 wt.% in CBP	4.0	4.94/2.98/2.60	1.43/1.17/0.41	0.99/0.84/0.25	2,004	(0.63, 0.33)
BTDF-TTPA	4.6	0.83/0.11/0.09	0.40/0.06/0.03	0.32/0.06/0.02	147	(0.70, 0.30)
BTDF-TtTPA	3.8	1.44/0.19/0.16	0.60/0.09/0.04	0.35/0.06/0.02	163	(0.71, 0.29)

a *At the luminance of 1 cd m^−2^*.

b *At the current density of @1 mA cm^−2^*.

Moreover, exciton utilization efficiency (η_s_) of the devices can be calculated according to the following equation:

(1) $$\begin{array}{l} EQE=\left(\gamma \times \eta_{s}\times \phi_{PL}\right)\times \eta_{out} \end{array}$$

where γ is the ideal recombination efficiency of the injected holes and electrons under electrical excitation (≈100%); η_out_ is the light out-coupling efficiency (≈0.2); ϕ_PL_ is the intrinsic photoluminescence efficiency of the emitters. η_s_s of 34.8% and 54.4% were estimated for the doped devices based on BTDF-TTPA and BTDF-TtTPA, respectively. For the non-doped devices, η_s_s of 66.9 and 97.3% were achieved for BTDF-TTPA and BTDF-TtTPA, respectively. Both are exceeding the limit of the radiative exciton ratio of 25% for traditional fluorescence OLEDs, strongly proving the contribution of triplet excitons to the electroluminescence thanks to the ultrafast high-lying RISC process, namely, the “hot exciton” channels.

## Conclusion

In summary, aiming to exploit highly efficient organic deep-red to NIR OLEDs, we have designed and synthesized three novel BTDF-based emitters, and their photophysical, thermal and electrochemical properties were thoroughly investigated. By incorporating thiophene as π-bridge into the D-A-D skeleton, the emission peak was successfully red-shifted 67 nm without sacrifice in efficiency. Among the three emitters, BTDF-TTPA and BTDF-TtTPA exhibit deep-red emission that possesses the potential to fabricate deep-red to NIR fluorescence OLEDs. In the EL performance, a maximum EQE of 5.75% was achieved with a very low efficiency roll-off for the doped devices thanks to the large overlap of frontier molecular orbitals that induced high PLQY. Besides, non-doped devices have also been fabricated and a maximum EQE of 1.44% was obtained for the NIR emission with a peak of 690 nm. In short, the new thiophene-bridge planarization strategy provides us a successful avenue for designing high efficiency D-π-A and D-π-A-π-D organic deep-red to NIR emitters.

## Data Availability

All datasets generated for this study are included in the manuscript and/or the Supplementary Files.

## Author Contributions

WX, XC, and KL designed the whole work. WX and XC synthesized the investigated compounds. BL fabricated and characterized the electroluminescent devices. WX and ML carried out the theoretical calculation. WX, XT, and ZQ measured the photophysical, thermal, and electrochemical properties of the investigated compounds. WX wrote the paper with the support from XC and S-JS. All authors contributed to the general discussion.

### Conflict of Interest Statement

The authors declare that the research was conducted in the absence of any commercial or financial relationships that could be construed as a potential conflict of interest.
