# Supplementary material for: Thiophene Disubstituted Benzothiadiazole Derivatives: An Effective Planarization Strategy Toward Deep-Red to Near-Infrared (NIR) Organic Light-Emitting Diodes
Source: Front Chem. 2019 Apr 18;7:276. doi: 10.3389/fchem.2019.00276 (PMC6482244; doi:10.3389/fchem.2019.00276)
Supplement: Supplementary file 1 [file Table_1.DOCX]

**Supporting Information**

**Thiophene disubstituted benzothiadiazole derivatives: an effective planarization strategy toward deep-red to near-infrared (NIR) organic light-emitting diodes**

*Wentao Xie, Binbin Li, Xinyi Cai, Mengke Li, Zhenyang Qiao, Xiaohui Tang, Kunkun Liu, Cheng Gu, Yuguang Ma and Shi-Jian Su**

State Key Laboratory of Luminescent Materials and Devices and Institute of Polymer Optoelectronic Materials and Devices, South China University of Technology, Wushan Road 381, Guangzhou 510640, P. R. China

*** Correspondence:**Prof. Shi-Jian Su

E-mail: [mssjsu@scut.edu.cn](mailto:mssjsu@scut.edu.cn)

**Contents**

1. **General information**
2. **Device fabrication and characterization**
3. **Synthesis**
4. **Theoretical calculation**
5. **Photophysical properties**
6. **Thermal properties**
7. **Energy level**
8. **OLED characterization**
9. **General information**

^1^H-NMR and ^13^C-NMR spectra were measured on Bruker NMR spectrometer (Germany) operating at 600 and 150 MHz, respectively, with tetramethylsilane (TMS) as the internal standard. All the data were recorded by dissolving the samples in deuterated chloroform (CDCl_3_) and measured at room temperature. Matrix-assisted laser desorption/ionization-time of flight (MALDI-TOF) mass spectrum was obtained using an AXIMA-CFRTM plus instrument for confirming the molecular weight. Thermogravimetry (TG) measurement was performed on Netzsch TG 209 at a heating rate of 10 ℃ min^-1^ under a nitrogen flow atmosphere. Differential scanning calorimetery (DSC) analysis was obtained on Netzsch DSC 209 under a nitrogen flow atmosphere at three heating and cooling cycles with a heating rate of 10 ℃ min^-1^ and a cooling rate of 20 ℃ min^-1^, respectively. Cyclic voltammetry (CV) measurement was performed by using the CHI-600D electro-chemical work station with platinum wire as counter electrode, a glssy carbon electrode as working surface and Ag/AgCl as reference electrode at a scanning rate of 50 mV s^-1^ in nitrogen saturated 0.1 mol L^-1^ n-Bu_4_NPF_6_ acetonitrile and dichloromethane solution. Ultra-violet to visible absorption (UV-vis) spectra were measured using Perkin-Elmer Lambda 950-PKA UV-Vis, while photoluminescence (PL) spectra were recorded by FluoroMax-4 spectrofluorometer (Horiba Jobin Yvon). Photoluminescence quantum yield (PLQY) of both the solutions and films were measured by utilizing an integrating sphere of Hamamatsu absolute PLQY spectrometer (C11347-11). Transient PL decay was evaluated with 280 nm LED excitation source using Quantaurus-Tau fluorescence lifetime measurement system (C11367-03, Hamamatsu Photonics Co., Japan).

1. **Device fabrication and characterization.**

Glass substrates pre-coated with a 95-nm-thin layer of indium tin oxide (ITO) with a sheet resistance of 10 Ω per square were thoroughly cleaned in ultrasonic bath of tetrahydrofuran (THF), isopropyl alcohol (IPA), detergent, deionized water, and isopropyl alcohol (IPA) and treated with O_2_ plasma for 10 min in sequence. Organic layers were deposited onto the ITO-coated glass substrates by thermal evaporation under high vacuum (~10^−5^ Pa). Cathode, consisting of a 1-nm-thin layer of LiF followed by a 100-nm-thin Al layer, was patterned using a shadow mask with an array of 3 mm × 3 mm openings. Deposition rates are 1−2 Å s^-1^ for organic materials, 0.1 Å s^-1^ for LiF, and 6 Å s^-1^ for Al, respectively. Electroluminescence (EL) spectra were recorded by an optical analyzer, Photo Research PR745. The current density and luminance versus driving voltage characteristics were measured by Keithley 2420 and Konica Minolta chromameter CS-200. External quantum efficiency (EQE) was calculated from the luminance, current density, and EL spectrum, assuming a Lambertian distribution.

1. **Synthesis:**

All solvents and reagents were purchased from commercial sources and used as received without futher purification. The synthetic routes of the targe compounds are outlined as below.

**Figure S1.** The synthetic routes of the targe compounds.

***Synthesis of compound 5,6-difluoro-4,7-di(thiophen-2- yl)benzo[c][1,2,5]thiadiazole (3)***

To a solution of 4,7-dibromo-5,6-difluorobenzo[c][1,2,5]thiadiazole (**1**) (464 mg, 1.41 mmol), Pd_2_(dba)_3_ (177.5 mg, 0.194 mmol) and P(*o*-tol)_3_ (100 mg, 0.329 mmol) in 50 mL tetrahydrofuran (THF) was added tributyl (thiophen-2-yl) stannane (**2**) (1175 mg, 3.15 mmol) under a N_2_ gas, and then the reaction mixture was heated and refluxed overnight. After the reaction mixture was cooled to room-temperature, the resulting mixture was quenched with deionized water and followed by extraction with dichloromethane (DCM) three times, the combined organic phase was then washed with deionized water three times. The organic solution was dried over anhydrous MgSO_4_ and filtered, and finally the solvent was removed by distillation under reduced pressure. Orange solid was obtained with 361 mg in 77 % yield. ^1^H NMR (600 MHz, CDCl_3_) δ (ppm) 8.30 (d, *J* = 3.8 Hz, 2H), 7.62 (d, *J* = 5.1, 1.0 Hz, 2H), 7.31–7.26 (m, 2H).

***Synthesis of compound 4,7-bis(5-bromothiophen-2-yl)-5,6-difluorobenzo[c]-[1,2,5]thiadiazole (5)***

5,6-Difluoro-4,7-di(thiophen-2-yl)benzo[c][1,2,5]thiadiazole (**3**) (713 mg, 2.12 mmol) was dissolved in 50 mL anhydrous THF and stirred at 0 °C for 30 min. N-Bromosuccinimide (**4**) (1.2 g, 6.74 mmol) was then added in portions. The reaction mixture was stirred at room temperature for 12 h. The resulting mixture was quenched with deionized water, the reaction mixture was extracted with DCM. The combined red layer was dried over MgSO_4_ and concentrated. The crude product was purified by column chromatography with petroleum ether (PE)/DCM (1:1, v/v). Orange solid was obtained with 1.05 g in 86.2 % yield. ^1^H NMR (600 MHz, CDCl_3_, δ (ppm) 8.04 (d, *J* = 4.1 Hz, 2H), 7.22 (d, *J* = 4.1 Hz, 2H).

***Synthesis of compound 4,4'-(5,6-difluorobenzo[c][1,2,5]thiadiazole-4,7-diyl)bis(N,N-diphenylaniline) (BTDF-TPA)***

N,N-Diphenyl-4-(4,4,5,5-tetramethyl-1,3,2-dioxaborolan-2-yl)aniline (**6**) (821 mg, 2.21 mmol) and 4,7-dibromo-5,6-difluorobenzo[c][1,2,5]thiadiazole (280 mg, 0.85 mmol) were dissolved into 120 mL toluene, 50 mL ethanol and 40 mL aqueous K_2_CO_3_ solution (2 M) in a 250 mL round-bottom flask. After degassing with nitrogen gas for 15 min, Pd(PPh_3_)_4_ (49 mg, 0.043 mmol) was added quickly and further degassed with nitrogen gas for 10 min. The reaction mixture was then heated to 85 ℃ and stirred vigorously for 18 hours. After the reaction system was cooled to room temperature, the solvent was removed in vacuum and the mixture was extracted with DCM. The collected organic phase was further washed by deionized water three times, followed by saturated brines three times as well, the organic solution was dried over anhydrous MgSO_4_. Further purification by column chromatography on silica gel with dichloromethane as the eluents affords 500 mg orange-red solids in 89% yield. ^1^H NMR (600 MHz, CDCl_3_) δ (ppm) 7.73 (d, *J* = 8.7 Hz, 4H), 7.31 (t, *J* = 3.8, 1.9 Hz, 8H), 7.25 – 7.17 (m, 12H), 7.09 (dd, *J* = 10.5, 4.2 Hz, 4H); ^13^C NMR (130 MHz, CDCl_3_) δ (ppm) 151.36, 151.20, 150.66, 150.63, 149.32, 149.15, 148.48, 147.25, 131.34, 129.44, 125.30, 123.67, 123.26, 121.82, 117.87, 117.83, 117.79, 117.75, 77.27, 77.01, 76.76, 29.78, 29.33; MALDI-TOF: calculated: 658.77; found: 658.1193.


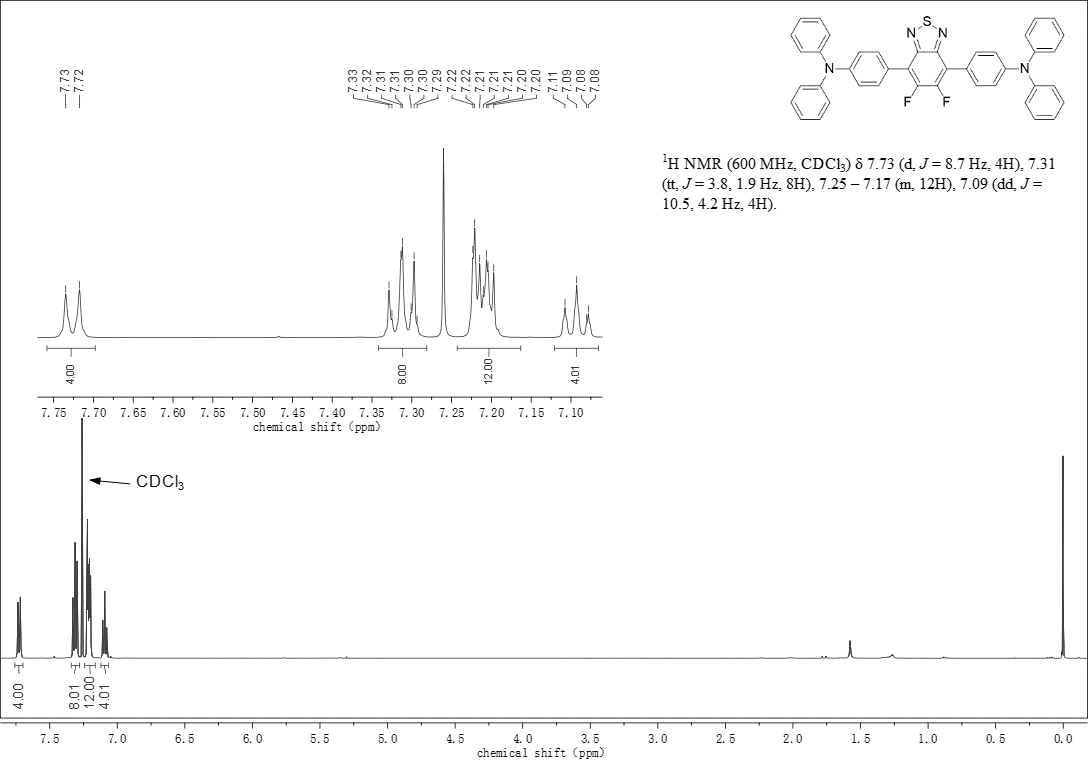


1. ^1^H-NMR spectrum of compound BTDF-TPA in deuterated CDCl_3_ solvent.


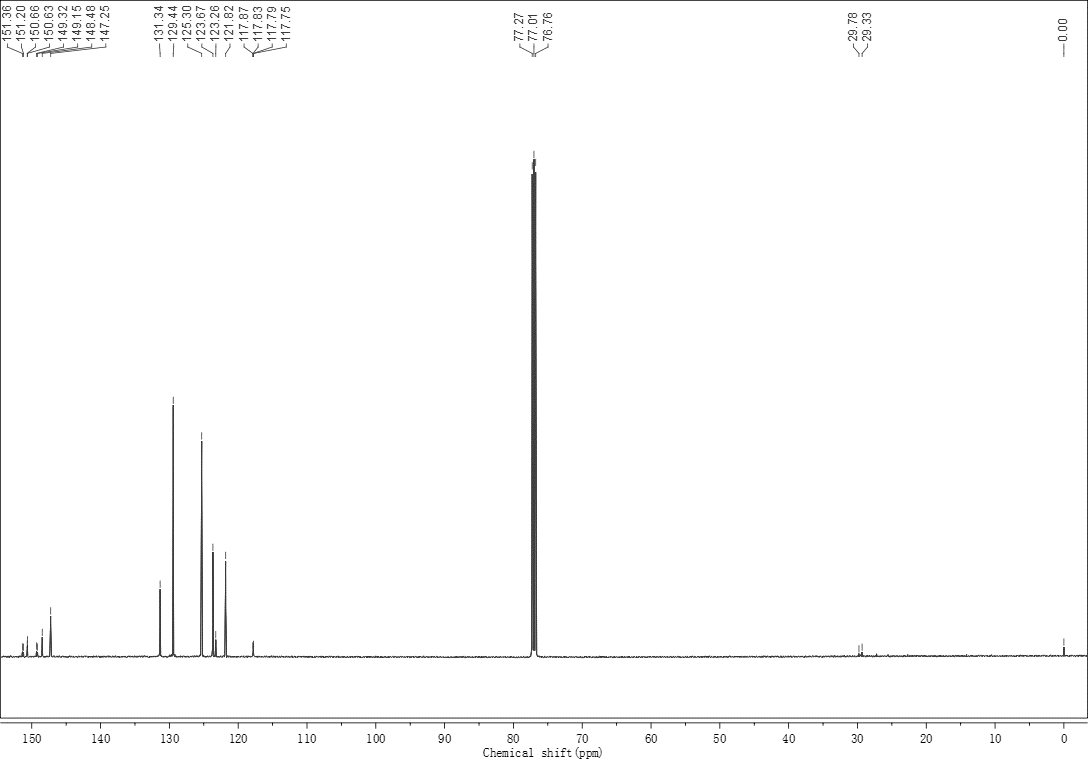


b) ^13^C-NMR spectrum of compound BTDF-TPA in deuterated CDCl_3_ solvent.

***Synthesis of compound 4,4'-((5,6-difluorobenzo[c][1,2,5]thiadiazole-4,7-diyl)bis(thiophene-5,2-diyl))bis(N,N-diphenylaniline) (BTDF-TTPA)***

BTDF-TTPA was synthesized in the similar procedure of BTDF-TPA, with 4,7-bis(5-bromothiophen-2-yl)-5,6-difluorobenzo[c][1,2,5]thiadiazole (**5**) (250 mg, 0.506 mmol) instead of 4,7-dibromo-5,6-difluorobenzo[c][1,2,5]thiadiazole. Further purification by column chromatography on silica gel with dichloromethane as the eluents affords 380 mg deep red solids in 91% yield. ^1^H NMR (600 MHz, CDCl_3_) δ (ppm) 8.27 (d, *J* = 3.8 Hz, 2H), 7.60 (d, *J* = 8.5 Hz, 4H), 7.38 (d, *J* = 3.7 Hz, 2H), 7.29 (t, *J* = 6.3 Hz, 8H), 7.09 (d, *J* = 35.2, 22.4, 14.1 Hz, 16H); MALDI-TOF: calculated: 823.01; found: 823.0559.

The low solubility of BTDF-TTPA hinders the measurement of ^13^C NMR spectrum even in chloroform solution.


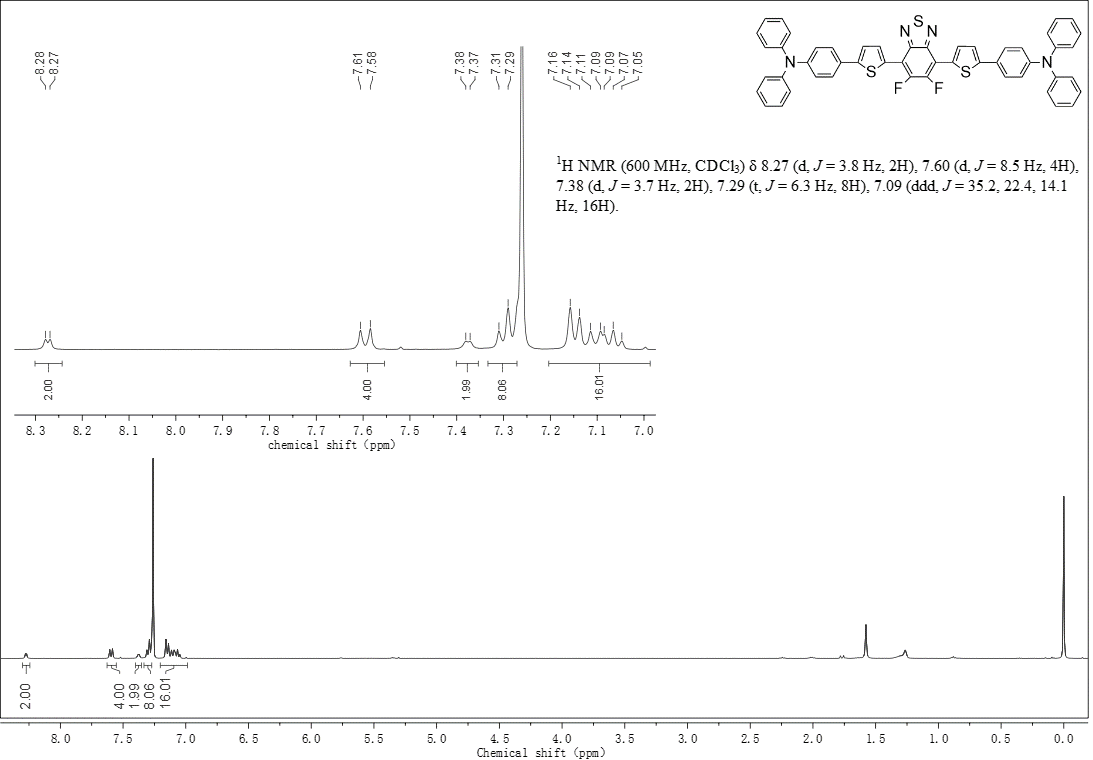


1. ^1^H-NMR spectrum of compound BTDF-TTPA in deuterated CDCl_3_ solvent.

***Synthesis of compound 4,4'-((5,6-difluorobenzo[c][1,2,5]thiadiazole-4,7-diyl)bis(thiophene-5,2-diyl))bis(N,N-bis(4-(tert-butyl)phenyl)aniline) (BTDF-TtTPA)***

BTDF-TtTPA was synthesized in the similar procedure of BTDF-TTPA, with 4-(*tert*-butyl)-N-(4-(*tert*-butyl)phenyl)-N-(4-(4,4,5,5-tetramethyl-1,3,2-dioxaborolan-2-yl)phenyl)aniline (**7**) (1001 mg, 2.07 mmol) instead of N,N-diphenyl-4-(4,4,5,5-tetramethyl-1,3,2-dioxaborolan-2-yl)aniline (**6**). Further purification by column chromatography on silica gel with dichloromethane as the eluents affords 830 mg deep red solids in 79% yield. ^1^H NMR (600 MHz, CDCl_3_) δ (ppm) 8.25 (t, *J* = 6.9 Hz, 2H), 7.56 (d, *J* = 8.6 Hz, 4H), 7.34 (d, *J* = 4.0 Hz, 2H), 7.29 (d, *J* = 8.6 Hz, 8H), 7.07 (d, *J* = 8.3, 4.2 Hz, 12H), 1.33 (s, 36H); ^13^C NMR (130 MHz, CDCl_3_) δ (ppm) 148.25, 146.22, 144.61, 126.82, 126.67, 126.16, 124.36, 122.43, 77.27, 77.22, 77.01, 76.76, 34.35, 31.44, 29.78, 29.33, 27.22; MALDI-TOF: calculated: 1047.44; found: 1047.2676.


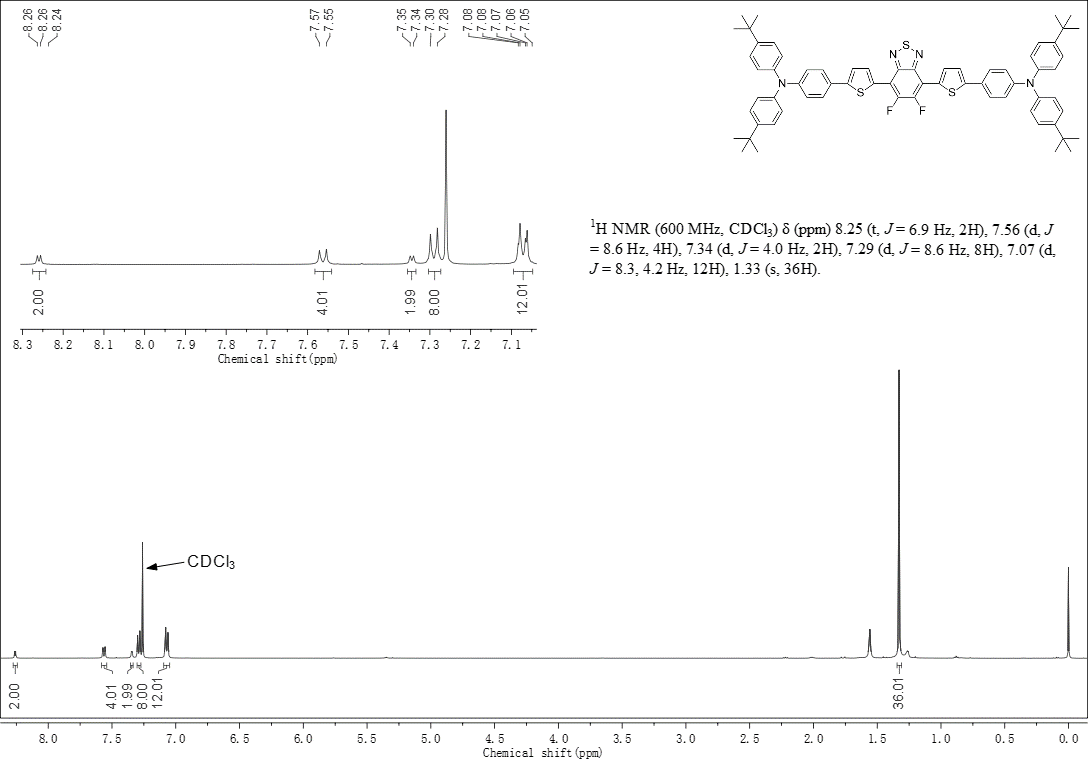


1. ^1^H-NMR spectrum of compound BTDF-TtTPA in deuterated CDCl_3_ solvent.

**
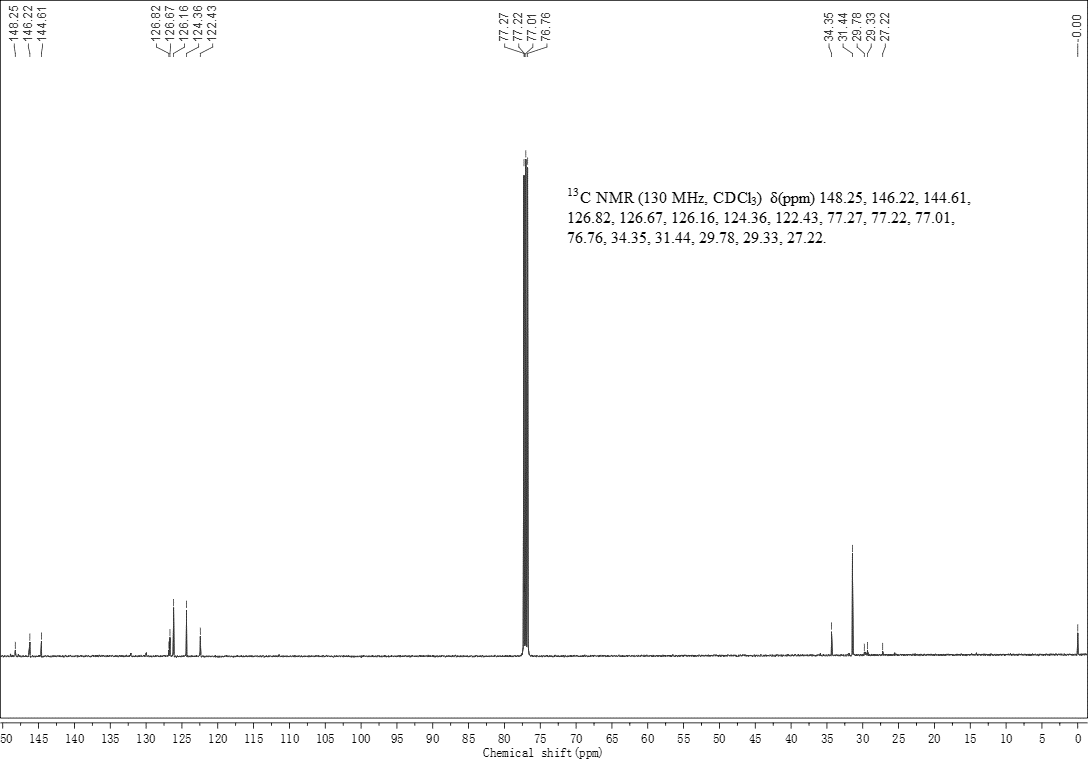
**

e) ^13^C-NMR spectrum of compound BTDF-TtTPA in deuterated CDCl_3_ solvent.

1. **Theoretical calculation**

**Figure S2.** NTOs of S_1_ state, T_1_ state and T_2_ state for these three molecules. Herein, *f* represents the oscillator strength, and the percentage weights of hole-particle are given.

1. **Photophysical properties**

**Figure S3.** UV-vis absorption and PL spectra of the BTDF-TTPA and BTDF-TtTPA neat films.

**Figure S4.** PL spectra of BTDF-TTPA and BTDF-TtTPA 1 wt.% doped in a common host CBP.

**Figure S5.** Transient PL decays of these investigated compounds in dilute toluene solution, 1 wt.% doped films in CBP and neat films.

| Table S1. Photophysical properties of the investigated materials in toluene, doped film and neat film. | | | | | |
| --- | --- | --- | --- | --- | --- |
|  | λ_em_ (nm) | τ_F_ (ns) | 𝜙_F_ | k_r_ (×10^8^ s^-1^) | k_nr_ (×10^8^ s^-1^) |
| BTDF-TPA in toluene | 560 | 1.28 | 95.2 | 7.4 | 0.4 |
| BTDF-TTPA in toluene | 630 | 0.96 | 85.8 | 8.9 | 1.1 |
| BTDF-TtTPA in toluene | 645 | 1.10 | 82.9 | 7.5 | 1.5 |
| BTDF-TTPA 1 wt.% in CBP | 625 | 4.11 | 82.7 | 2.0 | 0.4 |
| BTDF-TtTPA 1 wtl% in CBP | 635 | 4.21 | 45.4 | 1.1 | 1.4 |
| BTDF-TTPA neat film | 665 | 1.14 | 6.2 | 0.54 | 8.3 |
| BTDF-TtTPA neat film | 665 | 1.17 | 7.4 | 0.63 | 7.9 |

λ_em_ = emission maximum; 𝜙_F_ = absolute quantum yields determined with a calibrated integrating sphere system; k_r_ and k_nr_ were obtained by: k_r_=Ф_F_/τ_F_; τ_F_^-1^_=_ k_r_ + k_nr_.

**Lippert-Mataga calculation**

The properties of ground state (S_0_) and the lowest singlet excited state (S_1_) can be better understood through solvatochromic experiment. We use the Lippert-Mataga equation to explore the influence of solvent environment on the optical properties of these materials. The interaction between the solvent and the dipole moment of solute can be described by the model:

$$\mathrm{hc}\left( v_{a}-v_{f} \right)=hc\left( v_{a}^{0}-v_{f}^{o} \right)-\frac{2\left( \mu_{e}-\mu_{g} \right)}{a^{3}}f(\varepsilon,n)$$

where ƒ is the orientational polarizability of the solvent, $v_{a}^{0}-v_{f}^{o}$ corresponds to the Stokes shift when ƒ is zero, $\mu_{e}$ is the excited state dipole moment, $\mu_{g}$ is the ground-state dipole moment; *a* is the solvent cavity (Onsager) radius, derived from the Avogadro number (N), molecular weight (M), and density (d=1.0 g/cm^3^); *ε* and *n* are the solvent dielectric and the solvent refractive index, respectively; *f* (*ε*, *n*) and *a* can be calculated respectively as follows:

$$f(\varepsilon,n)=\frac{\varepsilon-1}{2\varepsilon+1}-\frac{n^{2}-1}{2n^{2}+1} , a={(\frac{3M}{4N\pi d})}^{1/3}$$

The detailed data are listed in Table S2.

| Table S2. Detailed photophysical data of the investigated materials in different polar solvents. | | | | | | | | | | |  |  |  |
| --- | --- | --- | --- | --- | --- | --- | --- | --- | --- | --- | --- | --- | --- |
| solvents | *f* ($\varepsilon,n$) | BTDF-TPA | | | BTDF-TTPA | | | | | BTDF-TtTPA | | | |
|  |  | λ_abs_  (nm) | λ_em_  (nm) | ν_a_-ν_f_  (cm^-1^) | | λ_abs_  (nm) | λ_em_  (nm) | ν_a_-ν_f_  (cm^-1^) | λ_abs_  (nm) | | | λ_em_  (nm) | ν_a_-ν_f_  (cm^-1^) |
| n-hexane | 0.0012 | 445 | 535 | 3780 | | 516 | 605 | 2851 | 528 | | | 618 | 2758 |
| carbon tetrachloride (CCl_4_) | 0.011 | 450 | 551 | 4073 | | 522 | 620 | 3028 | 534 | | | 629 | 2829 |
| triethylamine (TEA) | 0.048 | 448 | 548 | 4073 | | 521 | 618 | 3012 | 531 | | | 630 | 2959 |
| butyl ether (DBE) | 0.096 | 445 | 556 | 4486 | | 522 | 625 | 3157 | 532 | | | 640 | 3172 |
| isopropyl ether (IPE) | 0.145 | 443 | 560 | 4716 | | 522 | 637 | 3459 | 529 | | | 650 | 3518 |
| ethyl ether (DEE) | 0.167 | 441 | 565 | 4977 | | 514 | 641 | 3855 | 528 | | | 649 | 3531 |
| ethyl acetate (EA) | 0.200 | 436 | 593 | 6072 | | 514 | 654 | 4165 | 523 | | | 660 | 3968 |
| tetrahydrofuran (THF) | 0.210 | 440 | 594 | 5892 | | 518 | 659 | 4131 | 527 | | | 670 | 4050 |
| dichloromethane (DCM) | 0.218 | 443 | 613 | 6260 | | 518 | 666 | 4290 | 527 | | | 677 | 4204 |
| *N*,*N*-dimethylformamide (DMF) | 0.276 | 438 | 649 | 7423 | | 517 | 686 | 4765 | 526 | | | 686 | 4434 |
| acetone | 0.284 | 434 | 631 | 7194 | | 513 | 689 | 4979 | 521 | | | 699 | 4907 |
| acetonitrile (ACN) | 0.305 | 428 | 650 | 7980 | | 514 | 680 | 4750 | 516 | | | 707 | 5246 |

λ_abs_ = absorption maximum; λ_em_ = emission maximum; ν_a_-ν_f_ = the Stokes shift;


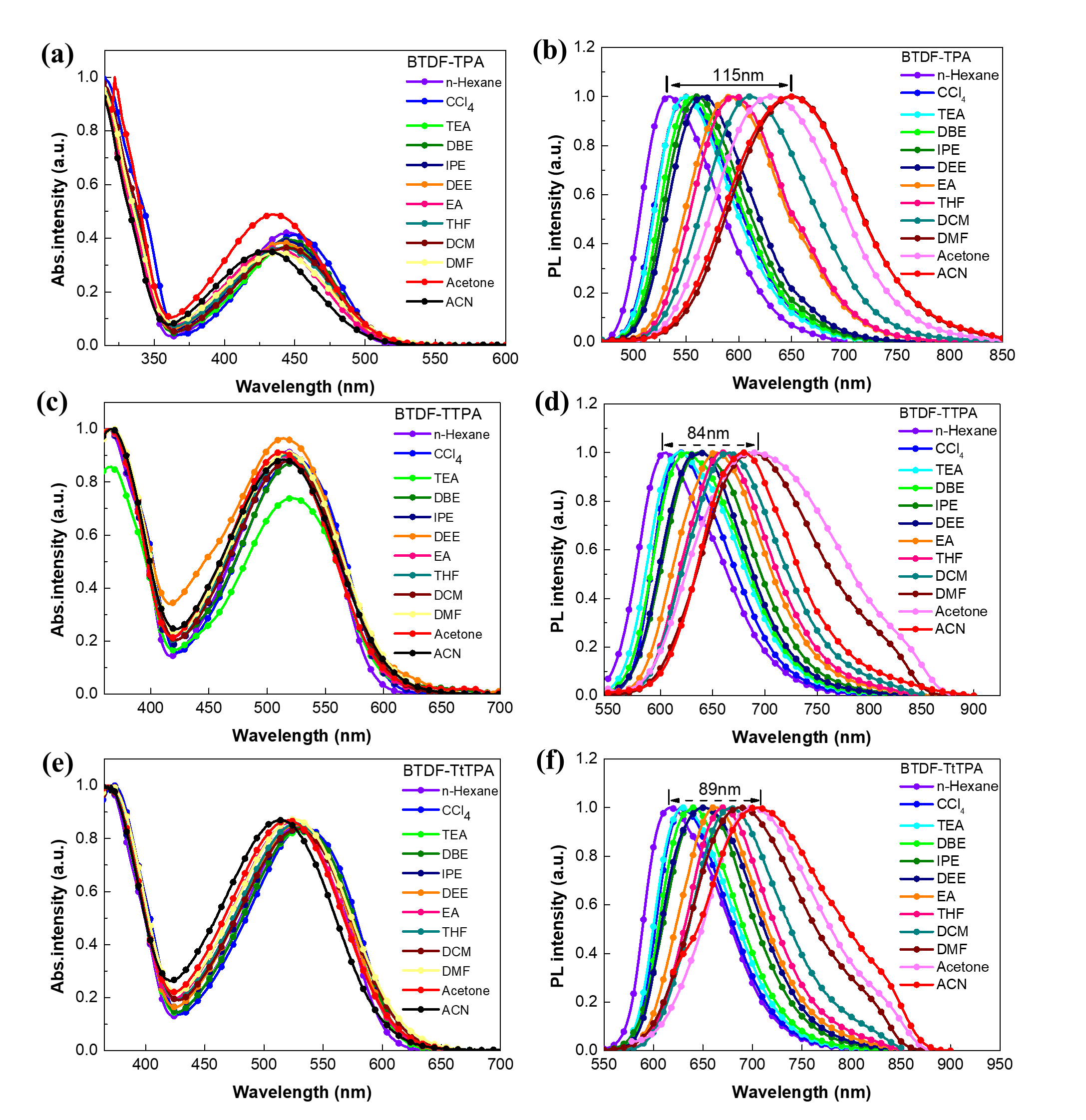


**Figure S6.** (a) and (b): UV-vis absorption and PL spectra of BTDF-TPA in different polar solvents; (c) and (d): UV-vis absorption and PL spectra of BTDF-TTPA in different polar solvents; (e) and (f): UV-vis absorption and PL spectra of BTDF-TtTPA in different polar solvents.

1. **Thermal Properties**

**Figure S7.** Thermal properties of these investigated compounds.

1. **Energy level**

**Figure S8.** Cyclic voltammograms (CV) of these investigated compounds.


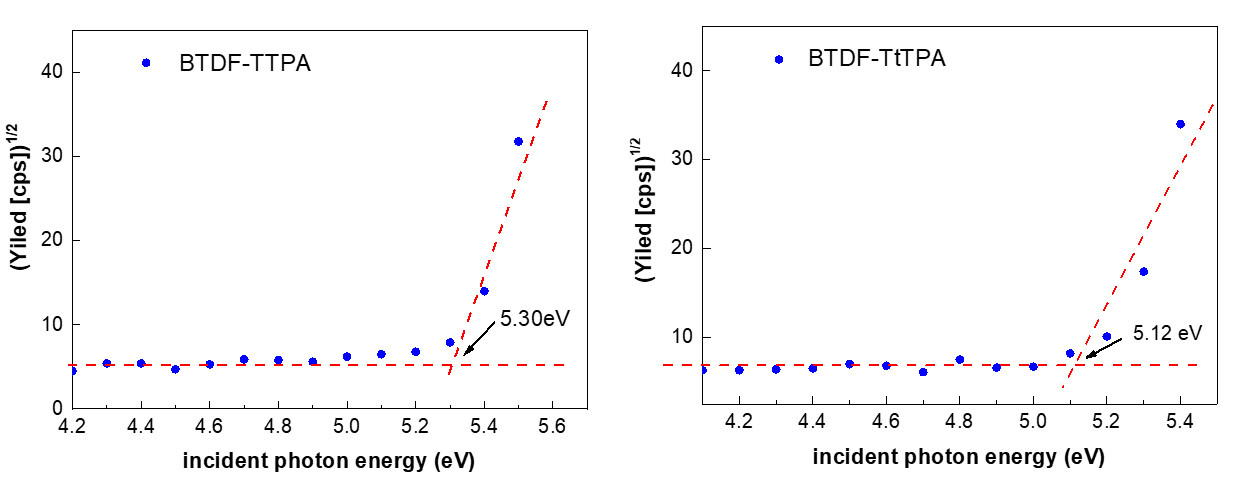


**Figure S9.** Photoelectron yield spectra (AC-3) of the vacuum evaporation prepared neat films of BTDF-TTPA and BTDF-TtTPA.

1. **OLED characterization**

**Figure S10.** Chemical structures of the materials used in device fabrication.
